# Grafting Neural Stem and Progenitor Cells Into the Hippocampus of Juvenile, Irradiated Mice Normalizes Behavior Deficits

**DOI:** 10.3389/fneur.2018.00715

**Published:** 2018-09-11

**Authors:** Yoshiaki Sato, Noriko Shinjyo, Machiko Sato, Marie K. L. Nilsson, Kazuhiro Osato, Changlian Zhu, Marcela Pekna, Hans G. Kuhn, Klas Blomgren

**Affiliations:** ^1^Center for Brain Repair and Rehabilitation, Institute of Neuroscience and Physiology, University of Gothenburg, Gothenburg, Sweden; ^2^Division of Neonatology, Center for Maternal-Neonatal Care, Nagoya University Hospital, Nagoya, Japan; ^3^Department of Obstetrics and Gynecology, Narita Hospital, Nagoya, Japan; ^4^Institute of Neuroscience and Physiology, University of Gothenburg, Gothenburg, Sweden; ^5^Department of Obstetrics and Gynecology, Mie University, Tsu, Japan; ^6^Department of Pediatrics, The Third Affiliated Hospital of Zhengzhou University, Zhengzhou, China; ^7^Department of Pediatric Oncology, Karolinska University Hospital, Stockholm, Sweden; ^8^Department of Women's and Children's Health, Karolinska Institutet, Stockholm, Sweden

**Keywords:** neural stem progenitor cells, irradiation, transplantation, grafting, learning deficits, developing brain, late effects

## Abstract

The pool of neural stem and progenitor cells (NSPCs) in the dentate gyrus of the hippocampus is reduced by ionizing radiation. This explains, at least partly, the learning deficits observed in patients after radiotherapy, particularly in pediatric cases. An 8 Gy single irradiation dose was delivered to the whole brains of postnatal day 9 (P9) C57BL/6 mice, and BrdU-labeled, syngeneic NSPCs (1.0 × 10^5^ cells/injection) were grafted into each hippocampus on P21. Three months later, behavior tests were performed. Irradiation impaired novelty-induced exploration, place learning, reversal learning, and sugar preference, and it altered the movement pattern. Grafting of NSPCs ameliorated or even normalized the observed deficits. Less than 4% of grafted cells survived and were found in the dentate gyrus 5 months later. The irradiation-induced loss of endogenous, undifferentiated NSPCs in the dentate gyrus was completely restored by grafted NSPCs in the dorsal, but not the ventral, blade. The grafted NSPCs did not exert appreciable effects on the endogenous NSPCs; however, more than half of the grafted NSPCs differentiated. These results point to novel strategies aimed at ameliorating the debilitating late effects of cranial radiotherapy, particularly in children.

## Introduction

The second most common type of childhood cancer is brain tumors, amounting to nearly one-third of all childhood cancers. The survival rates of pediatric brain tumor patients has improved in recent decades, and currently more than 80% of them survive (1). Although neurosurgery techniques and chemotherapy regimens have improved, radiation therapy (RT) is still an essential treatment modality not only for malignant brain tumors, but also for central nervous system (CNS) leukemia/lymphoma. However, RT causes both increased mortality and morbidity, in survivors of pediatric brain tumors (2). Cognitive impairments, as well as perturbed growth and puberty are some of the known late effects observed after RT. Moreover, it has been shown that the cognitive deficits are more severe in younger children after RT (3, 4), and the deficits increase progressively over time. It is unclear whether low doses of ionizing radiation (less than 0.5 Gy) administered to the CNS can cause cognitive impairment. One study claimed to find such effects (5), whereas a similar but larger study failed to do so (6). Ameliorating the late effects of RT will improve the quality of life of cancer survivors, whose prevalence is increasing, and particularly of pediatric cases, whose remaining life expectancy is long.

Irradiation (IR) causes injuries to many brain regions and cell types; however, the underlying pathogenesis is not clear. Neurogenesis persists throughout life in two regions, the subventricular zone (SVZ) and the dentate gyrus of the hippocampus. These regions harbor proliferating cells, and are therefore particularly susceptible to IR (7). Several reports suggest that injury to neural stem and progenitor cells (NSPCs) in the hippocampus can cause some of the late effects observed after IR (8–10), and the depletion of NSPCs induced by IR appears to be long-lasting, even after a single, moderate dose of IR (11, 12). The depletion is even aggravated over time (13). Currently, there are no established interventions after RT, but it was shown that memory training improved the attention and memory performance of children treated for medulloblastoma (14). Voluntary physical exercise increases the number of stem cells and enhances neurogenesis after IR of the young mouse brain, and at least partly normalizes IR-induced behavior changes (8, 15). In one study, human embryonic stem cells grafted into the hippocampus of adult immune-deficient rats improved their performance in a memory task after IR (16). On the other hand, grafting of syngeneic enteric neural stem cells (17) or syngeneic cerebral neural stem cells (18) into the hippocampus of irradiated mice caused local gliosis. Given the importance of inflammation-related signaling in the brain under both normal and pathological conditions, the aim of the present study was to explore whether grafting NSPCs into the hippocampus of immune-competent mice could ameliorate the deficits observed after IR at a young age in the absence of immunosuppressive treatment.

## Materials and methods

### Animals

All animal experimental protocols in the present study were approved by the Gothenburg committee of the Swedish Animal Welfare Agency (326-2009). C57BL/6J male pups with dams were purchased from Charles River (Sulzfeld, Germany) and maintained under a 12-h light/dark cycle with access to food and water *ad libitum*.

Six litters (6 pups/litter), in total 36 mice, were used for the study. Among them, 26 mice received IR on postnatal day 9 (P9). Three mice died during the IR procedure. Twenty-three irradiated mice were allocated into the NSPC (*n* = 13) or vehicle (*n* = 10) groups. All mice in the NSPC group were grafted with NSPCs and all in the vehicle group were injected with vehicle. However, 3 of 13 NSPC-grafted mice were excluded from analyses after histological evaluations as they were not grafted correctly (the cells were accidentally injected outside the hippocampus). All non-irradiated mice (*n* = 10) were allocated to the non-irradiated group and injected with vehicle. One IR NSPC mouse died during the IC chip insertion procedure, and one vehicle-treated mouse died before behavioral tests. Therefore, IntelliCage® was started with 13 NSPC-treated mice, 9 vehicle-treated mice and 10 non-irradiated mice. In the “Introduction 2” section in IntelliCage® (Supplementary Figure 2), when doors were closed, 1 NSPC-treated mouse and 2 vehicle-treated mice had not been able to learn how to do a “nosepoke” by the end of the section, and could hence not drink water. Therefore, these three mice were excluded from the “First corner” section. As 2 vehicle-treated mice died between the end of the IntelliCage® and the movement pattern analysis, the subsequent two behavioral tests were performed with 12 NSPC-treated mice, 7 vehicle-treated mice and 10 non-irradiated mice. One non-irradiated mouse died, and the sections for histological evaluations were made from the remaining mice (12 NSPC-treated mice, 7 vehicle-treated mice and 9 non-irradiated mice).

### Experimental procedures

IR was administered to P9 C57BL/6J mice, and NSPCs derived from the same mouse strain were injected into each hippocampus (in the right and left hemispheres) at P21. IR-Vehicle- treated mice received irradiation and were injected with vehicle instead of NSPCs. Non IR- Vehicle mice were only anesthetized, without irradiation, and received injections of vehicle (Supplementary Figure 1). Three months after grafting, the behavior of the mice was examined using three tests: IntelliCage®(P110-129), Movement pattern analysis (P162) and Sugar water (anhedonia) test (P166-170) (Supplementary Figure 1). After these behavioral tests, mice were sacrificed (P173, 5 months after grafting) (Supplementary Figure 1).

### Irradiation procedure

The IR procedure was performed as described earlier (18, 19). For IR, a linear accelerator (Varian Clinac 600C/D) with 4-MV nominal photon energy and a dose rate of 2.3 Gy/min was used. Nine-day-old mice were anesthetized with an intraperitoneal injection of tribromoethanol (Sigma-Aldrich, Stockholm, Sweden), placed in a prone position (head to gantry) on an expanded polystyrene bed. Both cerebral hemispheres of each animal were irradiated with a 2 × 2-cm asymmetrical radiation field. The source-to-skin distance was ~99.5 cm. The head was covered with a 1-cm tissue equivalent. A single absorbed dose of 8 Gy was administered to each mouse. Dose variation within the target volume was estimated to be ±5%. The entire procedure was completed within 10 min. After IR, pups were returned to their biological dams until weaning. Sham control animals were anesthetized but not subjected to IR. Using the LQ-model (20) and an α:β ratio of 3 for late effects in the normal brain tissue, an acute exposure of 8 Gy is equivalent to approximately 18 Gy when delivered in repeated 2-Gy fractions (21). This represents a clinically relevant dose, equivalent to the total dose used in treatment protocols for prophylactic cranial IR in selected cases of childhood acute lymphatic leukemia.

### Culture of NSPCs

NSPCs were kindly provided by Prof. Fred H. Gage (22), which were isolated from the whole brain of adult mice, except the cerebellum or the olfactory bulbs. NSPCs were cultured as described previously (18). Briefly, NSPCs were cultured and expanded as a monolayer in Dulbecco's Modified Eagle medium (DMEM)/nutrient mixture F-12 (1:1) (Invitrogen, San Diego, CA, USA) containing 1% N_2_ (Invitrogen), 20 ng/mL epidermal growth factor (Sigma-Aldrich, Saint Louis, MO, USA), 20 ng/mL basic fibroblast growth factor-2 (bFGF; BD Biosciences), and 5 μg/mL heparin (Sigma-Aldrich). Two days before grafting, BrdU was added to the medium at a final concentration of 1.25 μM. After collecting and washing, cells were suspended in DMEM containing 300 μg bFGF for grafting. More than 95% of the cells were viable as judged by a trypan blue exclusion test and more than 90% were BrdU-positive, as judged by immunostaining before grafting.

### NSPC grafting

NSPC grafting was performed as described previously (18). Mice were mounted onto a stereotactic head holder (Kopf Instruments, Tujunga, CA, USA) under anesthesia with isoflurane (Isoba® vet; Schering-Plough Corp., NJ, USA; 5% for induction, and 2% to 3% for maintenance) in the flat skull position. In order to graft NSPCs into the hippocampus, a 5-μL 26 gauge syringe (Innovative Labor Systeme, Stuetzerbach, Germany) attached to the holder was inserted according to the following coordinates: body weight < 9 g: 0.45 × (distance from lambda to bregma) mm posterior and ± 1.2 mm lateral to bregma, 3.0 mm deep from the skull surface; body weight ≥ 9 g, 0.42 × (distance from lambda to bregma) mm posterior and ± 1.3 mm lateral to bregma, 3.2 mm deep from the skull surface. Before the needle was inserted, a small hole was drilled in the proper position according to the above coordinates. Then, 1 × 10^5^ NSPCs in 2 μL DMEM were injected very slowly over a 2-min period, with a 4-min delay prior to removal of the syringe, and 2 min were allowed for syringe removal. No immunosuppressive drugs were administered.

### Tissue preparation

Mice were deeply anesthetized at P173 and intracardially perfusion-fixed using 0.9% NaCl followed by buffered formaldehyde (Histofix, Histolab, Göteborg, Sweden). Brains were removed and immersion-fixed in the same solution at 4°C for 24 h, and then, immersed in 30% sucrose for at least 2 days. Brains were cut sagittally at 30 μm on a sliding microtome in dry ice.

### Microscopy and immunohistochemistry

The following antibodies and final dilutions were used: anti-BrdU (1:500; AbD Seortec, Martinsried, Germany), goat anti-Sox2 (1:200; Santa Cruz Biotechnology, Santa Cruz, CA, USA), and rabbit anti-S100b (1:1000; Swant, Bellinzona, Switzerland). Immunoperoxidase detection of BrdU was performed as follows: free-floating sections were rinsed in Tris-buffered saline (TBS; 0.1 M Tris–HCl, pH 7.4/0.9% NaCl), and sections were then treated with 0.6% H_2_O_2_/TBS for 30 min, followed by incubation for 2 h in 50% formamide/2 × SSC (0.3 M NaCl, 0.03 M sodium citrate) at 65°C, rinsed in 2 × SSC, incubated for 30 min in 2 N HCl at 37°C, and rinsed in 0.1 M boric acid (pH 8.5). Incubation in TBS/3% donkey serum/0.1% Triton X (TBS++) for 30 min was followed by overnight incubation with mouse anti-BrdU. After rinsing in TBS, sections were incubated for 1 h with donkey anti-mouse-biotin (1:1,000 biotinylated donkey anti-mouse, Jackson ImmunoResearch Laboratories, West Grove, PA, USA) and then with avidin-biotin-peroxidase complex (Vectastain ABC Elite Kit, Vector Laboratories, Burlingame, CA, USA). This was followed by peroxidase detection for 5 min (0.25 mg/mL DAB, 0.01% H_2_O_2_, 0.04% NiCl). Immunoperoxidase detection of Sox2 was performed in the same manner with the proper secondary antibody, donkey anti-goat-biotin (1:1,000, Jackson ImmunoResearch Laboratories) as that of BrdU, except treatments for the formamide and HCl.

Triple immunofluorescence was performed as follows: free-floating sections were rinsed in TBS, and sections were incubated for 30 min in 2 N HCl at 37°C and rinsed in 0.1 M boric acid (pH 8.5). After several rinses in TBS, sections were incubated in TBS++ for 30 min, followed by a primary antibody cocktail containing rat anti-BrdU, goat anti-Sox2 and rabbit anti-S100b, for 24 h at 4°C. Sections were then rinsed in TBS, incubated with a cocktail of fluorochrome-labeled secondary antibodies for 2 h (1:1,000 donkey anti-rat Alexa 488, donkey anti-goat Alexa 546 and donkey anti-rabbit Alexa 647, Invitrogen Corporation, Carlsbad, CA, USA), rinsed again in TBS, and mounted on glass slides.

### Stereological quantification of cells

In each animal, every 12th section (typically 6 sections) containing a dorsal hippocampus was used to determine the total number of BrdU- and Sox2-positive cells in the granule cell layer (GCL) under light microscopy. These numbers were counted using stereology software (StereoInvestigator, version 6; MBF Bioscience, Williston, VT, USA). Cell counts were then multiplied with the series factor (12) to determine the total number of cells per GCL. In triple immunofluorescence, the percentage of each positive cell type (≥50 BrdU- or Sox2 -positive cells per animal) was assessed using a confocal microscope (Leica TCS SP2; Leica Microsystems, Wetzlar, Germany). Resulting percentages of each positive cell type were multiplied with the absolute number of BrdU- or Sox2-positive cells to calculate the absolute number of each cell type (23). GCL volume in P173 mice was calculated by measuring the area in every 12th section throughout the hippocampus, and the total sum of the areas traced for dorsal and ventral blades separately was multiplied by section thickness and series number to produce the entire GCL volume.

### Behavioral tests

#### IntelliCage®

IntelliCage® is a novel system for unbiased automated monitoring of spontaneous and learning behavior of mice in their home cage environment. Each cage had four corners, and each corner contained two water bottles. A door was located in front of each water bottle, and the door opened when a mouse touched it with its nose. Each cage housed maximum of 11 mice. Mice from the same litters were kept together in the same IntelliCage. Cage 1: 4 NSPC-treated, 3 vehicle-treated and 4 non-irradiated mice. Cage 2: 5 NSPC-treated, 3 vehicle-treated and 2 non-irradiated mice. Cage 3: 2 NSPC-treated, 2 vehicle-treated and 2 non-irradiated mice. Cage 4: 1 NSPC-treated, 1 vehicle-treated and 2 non-irradiated mice. Microchips (DataMars, Bedano, Switzerland) for identification were implanted s.c. in the dorsal neck of the mice on P54 under isoflurane anesthesia.

This behavioral test consisted of five sections (Supplementary Figure 2). The objective of the first two sections was for the mice to learn how to drink water. In the first section, “Introduction 1,” every mouse was permitted to drink in all corners, and cage doors were kept open to aid the mice in locating the water bottles. In the second section, “Introduction 2,” the doors were closed, and the mice, therefore, had to touch them with their noses (“nosepoke”) to open them and drink water. In the third section, “Corner training 1,” one corner was randomly allocated to each mouse. The mouse could open the door only in its allocated corner. The number of times a mouse opened the door in its allocated corner and/or tried to open it in the other corners were automatically recorded. In the next section, “Corner training 2,” the corner allocated in the previous section was changed randomly. Each mouse could access only its own allocated corner. In the final section, “Corner training 3,” the allocated corner was changed again.

#### Movement pattern analysis

The method and variables used for movement pattern analysis have been described in detail elsewhere (24, 25). Briefly, on the day of the experiment, mice were individually introduced into an unfamiliar open field arena and videotaped for 10 min in their inactive phase (lights on). Four arenas were simultaneously videotaped from above with a single CCD Monochrome video camera. The camera was connected to an S-VHS videocassette recorder. The arenas were made of black Plexiglas (l × w × h = 46 × 33 × 35 cm, respectively) rubbed with sandpaper and indirectly illuminated by bright light to avoid reflexes and shadows. The arena floors were covered with light gray gravel that had been previously exposed to other mice. The gravel was used to contrast the black color of the mice, and to act as an absorbent. It was exposed to other mice to saturate it with smells from multiple other individuals in order not to distract them to or from certain areas of the arena more than other areas. After completion of the experiment, videotapes were analyzed with the video tracking program EthoVision 3.1 (Noldus Information Technologies, Wageningen, Netherlands). For each sampling occasion, the program provided data on the position of the mouse as well as the animal's body area viewed by the overhead camera. The analysis resulted in a track record which described the animals' behavioral pattern during the observation period. Video tracking was performed at a sampling frequency of 12.5 Hz. Variables were calculated in a middle zone, defined as the central part located ≥6 cm from the arena walls, as well as in a border zone (< 6 cm from the arena walls). We evaluated the following variables: distance moved, number of stops made, and percent time spent in motion.

#### Sugar water (Anhedonia) test

This test spanned 4 days. Every mouse was housed individually, and normal water (Day 1 and 2) or 3% fructose water (Days 3 and 4) was supplied from the starting time of the night cycle after 6 h of water restriction. We measured the volume (weight) of water/sugar water each mouse drank per night. We then calculated the ratio of consumption (Day 3 + 4/Day 1 + 2) to evaluate preference for the fructose water. A decrease in fructose intake and preference over water is generally taken as a putative sign of anhedonia in rodents (26).

### Statistical analysis

All animal-related data are presented as mean ± standard error. ANOVA followed by Dunnett's *post-hoc* test was used to compare the three groups. A *p*-value of < 0.05 was considered to indicate a significant difference between compared mean values.

## Results

### Mortality and body weight gain

Thirty-six mice were used in the present study, but three mice died during anesthesia/irradiation, and 3 mice were excluded from analyses due to grafting failure. 1 NSPC-treated mouse died during the insertion of a microchip during anesthesia, and three vehicle-treated mice died during the observation period. Therefore, the mortality rates of each group after IR were 1/10 in NSPC-treated mice, 3/10 in vehicle-treated mice, and 1/10 in vehicle-treated non-irradiated mice.

**Figure 1:**
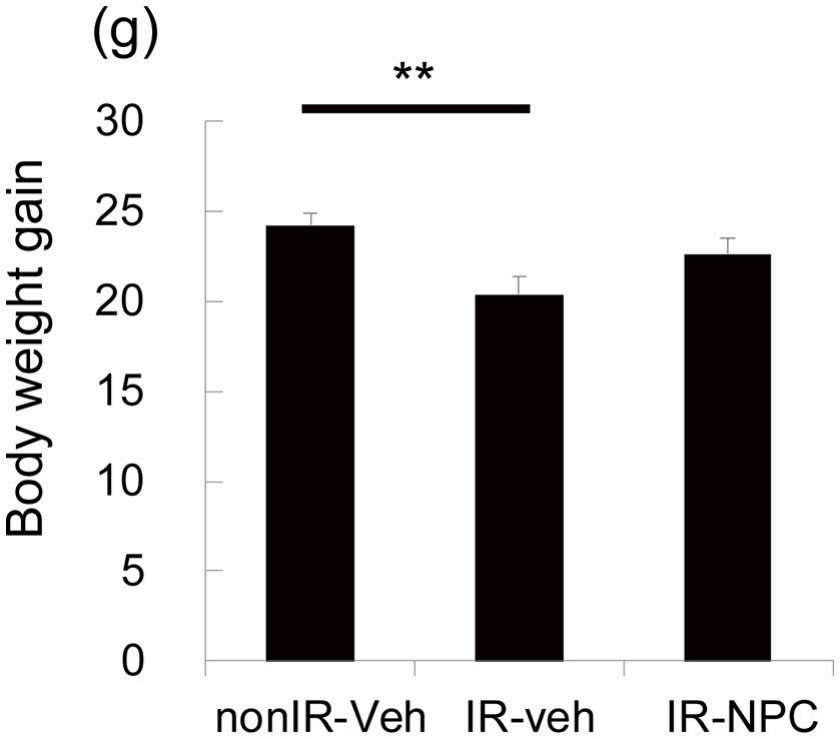
Body weight gain. The body weight gains from the point of grafting (P21) to sacrifice (P173) was significantly lower in vehicle treated mice (but not in NSPC-grafted mice) than those in treated non-irradiated mice (**p < 0.01).

The body weight gains from the point of grafting (P21) to sacrifice (P173) was calculated. The gain was significantly lower in irradiated vehicle-treated mice than in non-irradiated mice (Figure 1, *p* < 0.01), whereas irradiated, NSPC-grafted mice were not different from controls.

### Grafting NSPCs ameliorated the negative effects of IR on behavior

To examine the effects of grafting on behavior after IR, a single dose of 8 Gy cranial IR was administered at P9, and NSPCs were grafted onto both hippocampi at P21. Beginning 3 months after grafting, we examined the mice's behavior using three tests: IntelliCage®, movement pattern analysis (Open Field test), and sugar water preference (anhedonia test).

**Figure 2:**
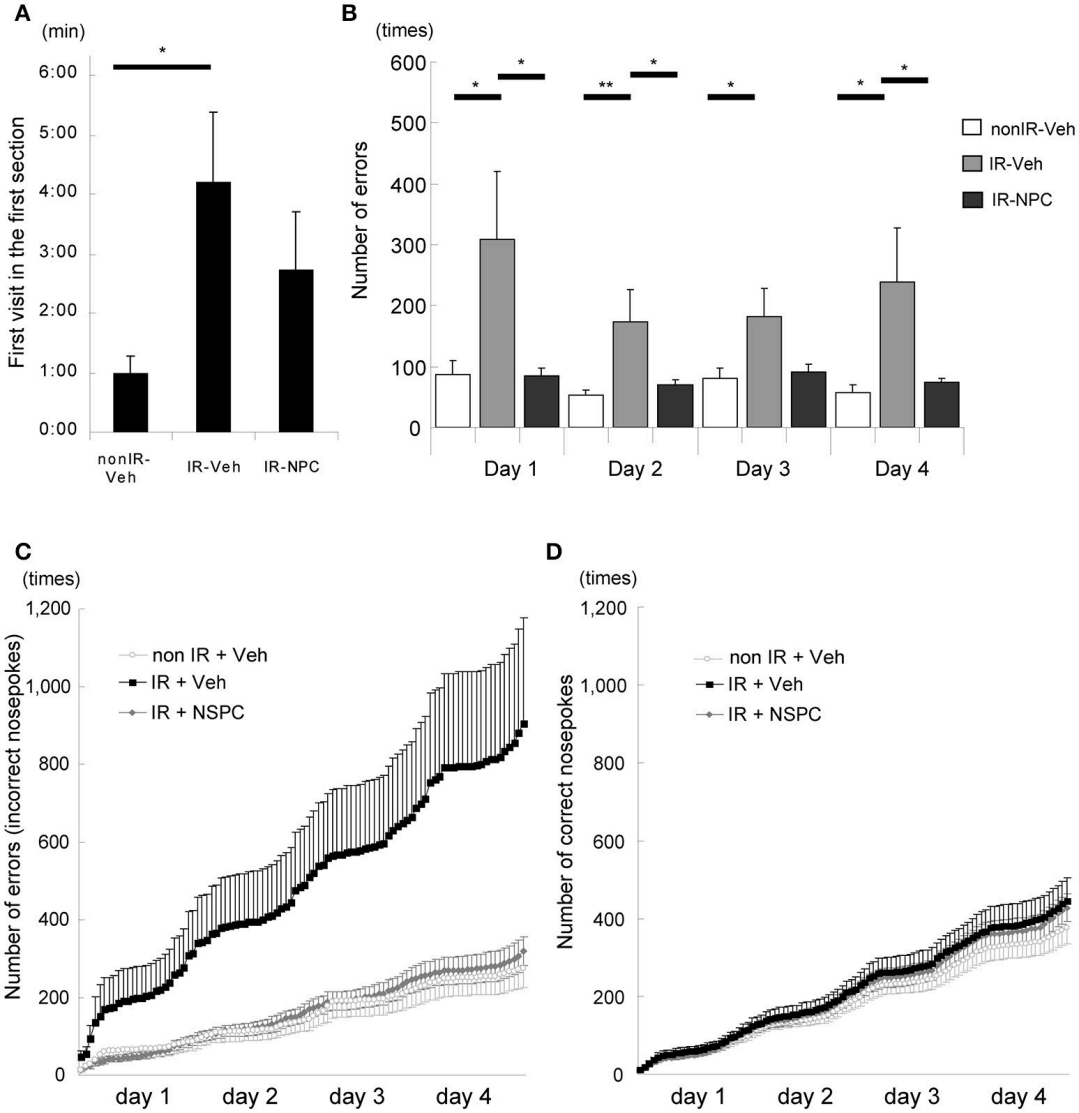
Learning deficit after irradiation (IR) (behavioral evaluation with Intellicage®). **(A)** First visit in the first section (Introduction 1). The time it took for irradiated mice injected with vehicle (IR-vehicle), but not with neural stem and progenitor cells (NSPCs) (IR-NSPC), to visit a corner was significantly longer than that for non-irradiated mice (non-IR-vehicle; *p < 0.05). **(B)** Number of errors (second corner). The number of times that each mouse tried to open the door in non-allocated corners (i.e., errors) was significantly higher for irradiated (IR-vehicle) than for irradiated and grafted (IR-NSPC) and non-irradiated (non IR-vehicle) mice (*p < 0.05, **p < 0.01). **(C)** Accumulated number of errors (second corner). The accumulated number of errors that each mouse made (trying to open a non-allocated door) in the IR-vehicle group was higher than that in the non IR-vehicle group, but that in the IR-NSPC group was almost identical to that in the non-IR-vehicle group. Open circle indicates the non-IR-vehicle group; closed square indicates the IR-vehicle group; and closed diamond indicates the IR-NSPC group. **(D)** Accumulated number of correct nosepokes (second corner). The accumulated number of correct nosepokes was almost identical between the three groups. Open circle indicates the non-IR-vehicle group; closed square indicates the IR-vehicle group; and closed diamond indicates the IR-NSPC group. Data represent the mean ± S.E.M.

IntelliCage® is a system for unbiased automated monitoring of spontaneous activity and learning of mice in their home cage environment (27–34). In the first session, we measured the time it took for each mouse to visit a corner, representing a combined measure of novelty-induced exploratory behavior and anxiety. Irradiated, vehicle-injected mice took four times longer to visit the first corner compared with non-irradiated mice (Figure 2A, *p* < 0.05), whereas irradiated, NSPC-grafted mice took an intermediate length of time (Figure 2A). The number of times that each mouse tried to open the door in non-allocated corners (errors) was examined. The number of errors in the second corner session (reversal learning) was significantly higher for irradiated, vehicle-injected mice than for irradiated, grafted mice (Figure 2B; Days 1, 2, and 4, *p* < 0.05) and non-irradiated mice (Figure 2B; Day 2, *p* < 0.01; Days 1, 3, and 4, *p* < 0.05). The accumulated number of errors during the second corner session (reversal learning), as registered when a mouse tried to open a door in a non-allocated corner, was higher for the IR + vehicle group than the other two groups (Figure 2C). Irradiated, vehicle-injected mice made several-fold more errors than non-irradiated or irradiated NSPC-grafted mice. Importantly, the number of correct door openings did not differ between groups (Figure 2D). Both this IR-induced learning deficit and the ameliorating effect of NSPC grafting were also apparent in the first and third corners (Supplementary Figures 3A,B). These results indicate that IR significantly impaired place learning and reversal learning, and grafting of NSPCs significantly ameliorated these deficits.

**Figure 3:**
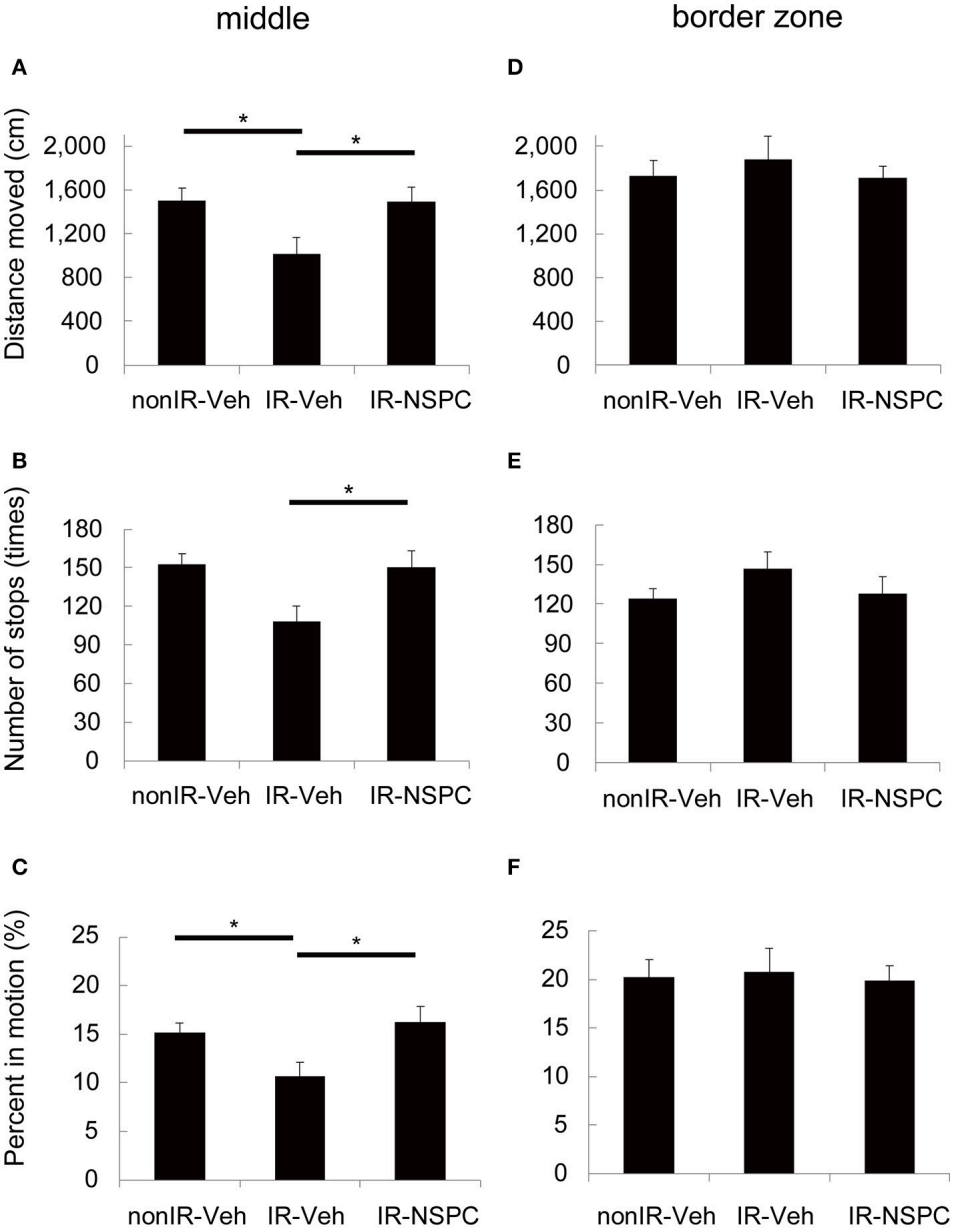
Movement pattern analysis. We evaluated the effect of irradiation (IR) and neural stem and progenitor cell (NSPC) grafting on the movement pattern of mice using the Open Field test. IR significantly decreased the distance moved **(A)** (*p < 0.05) and the number of stops made **(B)** (*p < 0.05) and tended to decrease the percent time spent in motion **(C)** in the middle. NSPCs grafting normalized the effect of IR on these variables **(A–C)** (*p < 0.05 vs. IR group). Neither IR nor NSPCs grafting changed any variables significantly in the border zone **(D–F)**. Data represent the mean ± S.E.M.

The effect of IR and NSPC grafting on movement pattern in the Open Field test was analyzed with respect to the distance moved, number of stops, and percent time in motion. IR significantly decreased the distance moved (Figure 3A, *p* < 0.05) and the number of stops made (Figure 3B, *p* < 0.05), and tended to decrease the time in motion (Figure 3C) in the middle of the arena. On the other hand, there were no significant differences in any variables in the border zone (Figures 3D–F). The irradiated mice appeared more “anxious” and avoided the middle of the arena, but this was normalized by the grafting of NSPCs. (Figures 3A–C, *p* < 0.05 vs. IR group).

**Figure 4:**
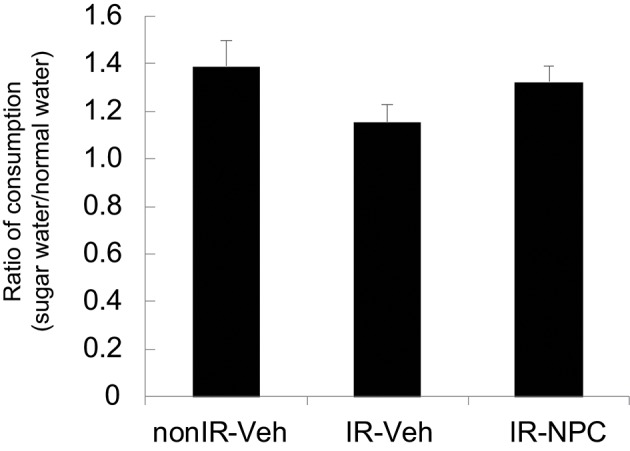
Sugar water preference (anhedonia test). The ratio of consumption [Day 3 + 4 (sugar water)/Day 1 + 2 (normal water)] was examined. Irradiated mice treated with vehicle, but not grafted mice, tended to consume less sugar water than did non-irradiated mice. Data represent the mean ± S.E.M.

We also applied the sugar water preference or anhedonia test. Irradiated mice treated with vehicle, but not NSPC-grafted mice, tended to consume less sugar water than did non-irradiated mice (Figure 4).

### The fate of grafted cells and the effect of irradiation and/or NSPC grafting on morphological change and endogenous neural stem cells

**Figure 5:**
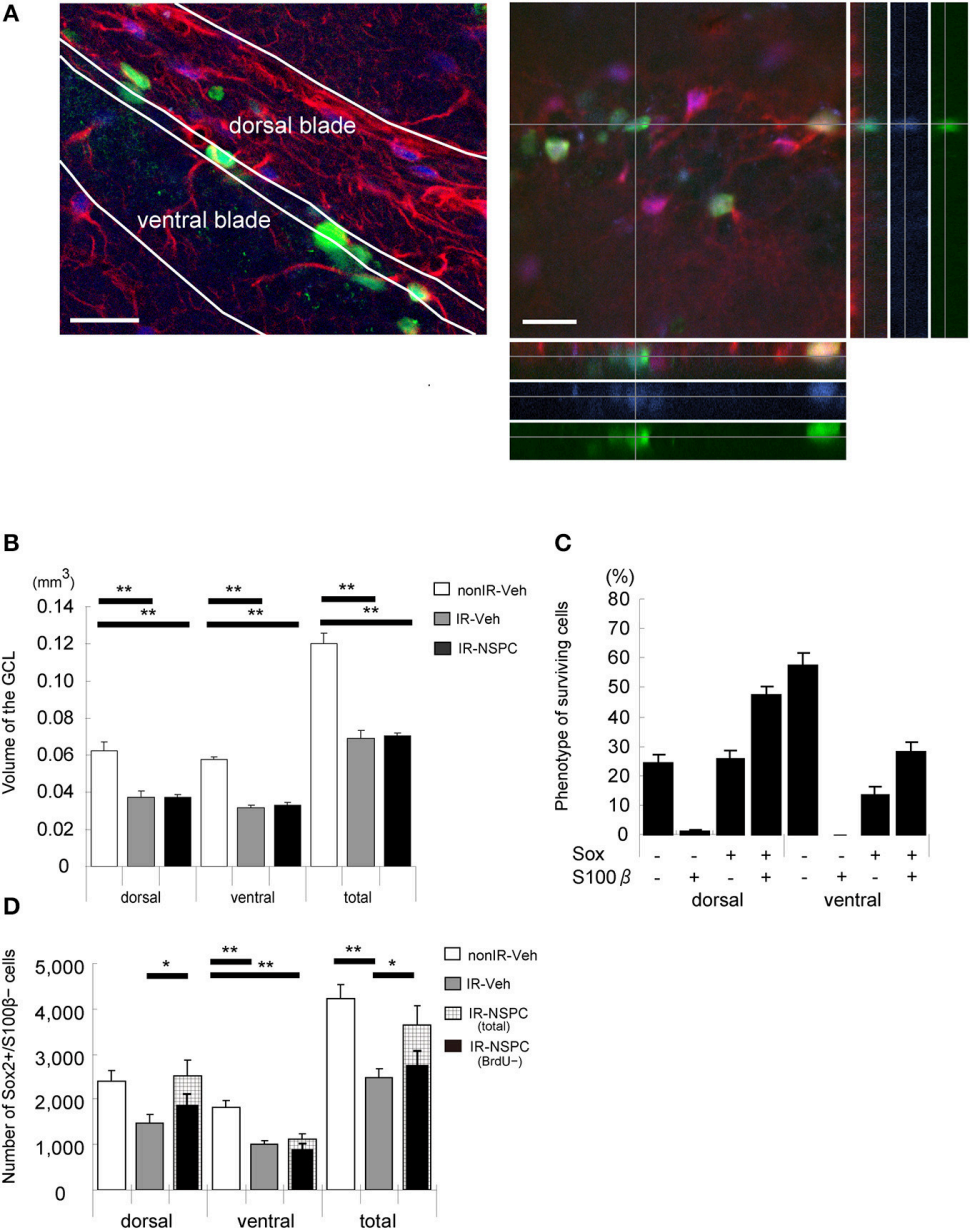
The effect of irradiation (IR) and grafting on the morphology and endogenous neural stem and progenitor cells (NSPCs) of the GCL and the fate of surviving cells (5 months after grafting). **(A)** Representative microphotograph of the hippocampus 5 months after grafting. Stained for BrdU (green), Sox2 (blue), and S100β (red). Note that surviving grafted cells remained in the whole GCL, and gliosis-like change existed in the dorsal blade of the GCL. A Z-stack was created to analyze BrdU+/Sox2+/S100β+ cells to verify that they were truly double- or triple-positive. Scale bar = 25 μm. **(B)** Volume of the GCL. There was a significant decrease in irradiated, vehicle-injected and grafted mice compared with non-IR mice (**p < 0.01). Volume of grafted mice was almost identical to that of vehicle-injected mice. **(C)** Phenotype of surviving cells. We evaluated the phenotype of surviving cells by staining for BrdU, Sox2, and S100β. Only 14% of BrdU-positive cells in the lower blade and 25% in the upper blade of the GCL were positive for Sox2 and negative for S100 β. **(D)** Total number of stem cells. We evaluated the number of stem cells by staining for Sox2 (positive) and S100β (negative). Total number of stem cells of irradiated vehicle-injected mice was significantly lower than that of non-irradiated mice. Grafted mice had almost the same number of stem cells in the upper blade as non-irradiated mice, but the number in the lower blade was reduced to that of irradiated vehicle-injected mice (**P < 0.01, *P < 0.05). We also evaluated the number of endogenous stem cells by staining for BrdU (negative), Sox2 (positive), and S100β (negative). Number of grafted mice was almost the same as that of irradiated vehicle-injected mice. Volume measurement and positive cell counting were conducted as described in the Materials and Methods section. Data represent the mean ± S.E.M.

We evaluated the number and fate of the grafted cells, the GCL size after grafting, and the effect of grafted NSPCs on endogenous neural stem cells. Five months after IR, 7,222 ± 455 BrdU-positive grafted cells per brain survived in the GCL; i.e., 3.6% of the total number of cells injected. Figure 5A shows a representative image of the hippocampus 5 months after grafting.

IR induces apoptosis of endogenous NSPCs and thereby arrests subsequent growth of the GCL in irradiated developing and juvenile brains (7). NSPC grafting did not increase the GCL volume in irradiated brains, in the dorsal or ventral blades (Figure 5B). The GCL consists mainly of tightly packed granule neurons, and the grafted cells hence did not seem to replace the granule cells that failed to develop after IR.

We estimated how many grafted NSPCs remained undifferentiated, using Sox2 and S100β staining, assuming that Sox2+/S100β− cells represent undifferentiated neural stem cells (double positive cells were astrocytes, whereas double negative cells could be neurons or oligodendrocytes) (35). In the ventral blade of the GCL, 14% of the grafted cells were Sox2+/S100β−, whereas 58% were negative for both Sox2 and S100β. In the dorsal blade, however, 26% of grafted cells were Sox2+/S100β−, and only 25% were negative for both Sox2 and S100β (Figure 5C). The total number of undifferentiated (Sox2+/S100β−) cells were counted in each GCL; i.e., both grafted and endogenous undifferentiated NSPCs (Figure 5D). IR reduced the number of undifferentiated NSPCs to almost half that observed in non-IR hippocampi in both the dorsal and ventral blades (Figure 5D, *p* < 0.01). Interestingly, NSPC grafting restored the number of undifferentiated NSPCs to control levels but only in the dorsal blade (Figure 5D). In the ventral blade, grafting did not affect the number of undifferentiated neural stem cells (Figure 5D). The normalized number of undifferentiated cells in the dorsal blade could be the result of grafted cells populating the subgranular zone (SGZ), grafted cells stimulating the proliferation and/or survival of endogenous neural stem cells, or a combination of the two. To differentiate between endogenous and grafted undifferentiated neural stem cells, we counted the number of BrdU-negative, undifferentiated (BrdU–/Sox2+/S100β−) cells; i.e., the number of endogenous, undifferentiated cells (Figure 5D). The number of endogenous, undifferentiated cells in irradiated, grafted mice was virtually identical to that of irradiated, non-grafted mice, although there was a tendency toward higher numbers in the dorsal blade of grafted mice (Figure 5D). Therefore, the normalized number of NSPCs in the dorsal blade of irradiated, grafted mice seems to result mainly from the grafted cells populating the SGZ and remaining undifferentiated.

## Discussion

In the present study, we have demonstrated that grafted NSPCs can survive in the GCL for at least 5 months, that IR caused behavioral abnormalities, including place learning deficits, and that NSPC grafting into the hippocampus could ameliorate or normalize the behavioral abnormalities induced by IR.

In a previous study, we showed that the survival of grafted NSPCs was significantly impaired and that neuronal differentiation was lower in irradiated than in non-irradiated brains when grafting was performed 24 h after IR (53% in irradiated brains vs. 84% in non-irradiated brains), whereas there were no significant differences when grafting occurred 1 week (64 vs. 66%) or 6 weeks (29 vs. 38%) after IR (18). These findings indicate that we should not graft NSPCs in this acute phase, soon after IR. Grafting cells before IR was deemed not useful, considering that proliferating neural stem cells are very susceptible to IR (7, 19, 11, 36), and a large portion of the grafted NSPCs would be killed by IR and handling the dead and injured cells would add to the burden of the tissue. Based on these findings, we grafted the NSPCs after the acute phase and observed an ameliorating effect of grafting on CNS complications after IR. However, it remains unknown whether grafting before or in the acute phase after IR also would exert such an effect.

Since proliferating neural stem cells are highly susceptible to IR and since a reduced number of proliferating NSPCs in the GCL of the dentate gyrus of the hippocampus can lead to learning/behavioral abnormalities later in life, we hypothesized that exogenous NSPCs could ameliorate the side effects of IR therapy by compensating for the lost cells. In addition, the younger the brain, the higher the number of differentiated neuronal cells after grafting (18). NSPC grafting may therefore be more effective in developing brains than in adult brains. In the present study, we showed that the NSPC grafting into the hippocampi of developing brains could ameliorate learning deficits and behavioral abnormalities. It is known that NSPCs secrete various neurotrophic factors and exert neuroprotective effects (37, 38). In several animal models of stem cell grafting for neurodegenerative diseases, including stroke, both morphological and functional recovery have been observed, even when only a few grafted cells survived. It was reported that grafted cells did not replace degenerated neuronal cells but provided trophic factors and/or suppressed the immune/inflammatory response that induces angiogenesis, neurogenesis, and/or neuroprotection (39). Grafted NSPCs or mesenchymal stem cells enhance proliferation of endogenous cells and neurogenesis in neurogenic regions including the hippocampus and SVZ (40). To elucidate the mechanism of the ameliorating effect of NSPC grafting after IR in the present study, we evaluated the trophic effect of the grafted NSPCs. Specifically, these effects were the volume of the GCL of the hippocampus, which is reduced after IR due to reduced growth (19, 31), and the number of endogenous neural stem cells, based on the number of Sox2-positive and S100β-negative cells and BrdU-negative cells. However, no trophic effects were observed in the present study, as judged by the virtually identical size of the GCL and number of endogenous neural stem cells in irradiated grafted mice were compared to those in irradiated vehicle-injected mice. On the other hand, in our previous study, 5 weeks after grafting at P21, more than 60% of surviving cells were neurons (18). The present study also showed that at 5 months after grafting, more than 50% of surviving cells in the ventral blade were negative for both Sox2 and S100β, which indicated that they adopted a neuronal or oligodendrocytic phenotype, although it was not confirmed with a neuronal marker like NeuN. Although the functionality of the surviving neurons differentiated from grafted NSPCs remains to be evaluated, it is possible that grafted cells replaced, to some extent, the IR-induced loss of NSPCs. This in turn may have led to amelioration of abnormalities after IR. Another possible explanation could be continuous production of growth and trophic factors by NSPCs, which over time leads to changes in connectivity and other functional aspects not related to the actual numbers of cells or their phenotypes.

Toward clinical applications of NSPC grafting for IR-induced learning and behavioral deficits, several issues remain to be addressed. One of these is grafting-induced astrogliosis. We previously showed that injection of NSPCs, but not the vehicle, into the hippocampus induced astrogliosis and reduced thickness of the dorsal blade of the GCL (18). Cell grafting can induce and promote glial scarring (17, 41) and upregulation of expression of chondroitin sulfate proteoglycans around the injection site (42). These cause impaired migration and neuritogenesis of grafted cells, leading to abortive host-graft integration. In a clinical application of NSPC grafting, some modification [e.g., inhibition of reactive astrocytes (43), enzymatic removal of chondroitin sulfate (44)], may be needed to circumvent this problem and enhance the therapeutic effect.

In the present study, we administered NSPCs directly into the hippocampus. In other studies of neuronal diseases, intravenous administration has also been used (45–47). Systemic administration may be advantageous, especially for diseases with multifocal and/or disseminated lesions such as multiple sclerosis. Although NSPCs administrated intravenously can migrate into lesions to some degree, the number of cells is limited, up to 13% (45), and NSPCs accumulate in organs without lesions (47). More recently, an intranasal route for administration of NSPCs has been developed (48–50). It was shown that intranasally administered stem cells can migrate into the brain. These alternative administration routes are less invasive than intracranial grafting but also less effective, and for clinical applications a balance between safety and efficacy must be established.

It may be argued that using postnatal day 9 mice corresponds to a very young human brain, developmentally (51), an age when cranial radiotherapy wouldn't be considered in a clinical setting, but the numbers of endogenous NSPCs and the rate of neurogenesis are higher at this relatively early age, so for the purpose of studying the effects of IR and NSPC grafting, it is useful for proof of principle, as demonstrated in earlier studies in both rats and mice (8, 11, 19). Another reason is that the survival of grafted cells is better in a younger brain, as noted in our previous study (18). Further studies are needed to show the treatment effect of grafting NSPCs with older mice.

In conclusion, our results indicate that NSPCs grafting into the hippocampus can ameliorate or normalize the impaired place learning as well as the altered movement pattern induced by IR. Although ethical, technical and other issues remain to be addressed prior to its clinical application, NSPC grafting is promising as a therapeutic technique. Further studies are needed to optimize the treatment and clarify the underlying mechanisms.

## Author contributions

YS, MS, and KO were actively involved in experiments. NS cultured NSPCs for experiments. MN and YS performed open field test. YS, CZ, HK, MP, and KB conceptualized and designed this study. YS, CZ, HK, and KB interpreted data in this study. YS drafted an initial manuscript, and NS, MS, KO, CZ, MP, HK, and KB revised it critically. All authors approved the final manuscript as submitted and agree to be accountable for all aspects of the work.

### Conflict of interest statement

The authors declare that the research was conducted in the absence of any commercial or financial relationships that could be construed as a potential conflict of interest.
