# Supplementary figures and images for: Grafting Neural Stem and Progenitor Cells Into the Hippocampus of Juvenile, Irradiated Mice Normalizes Behavior Deficits
Source: Front Neurol. 2018 Sep 11;9:715. doi: 10.3389/fneur.2018.00715 (PMC6141740; doi:10.3389/fneur.2018.00715)

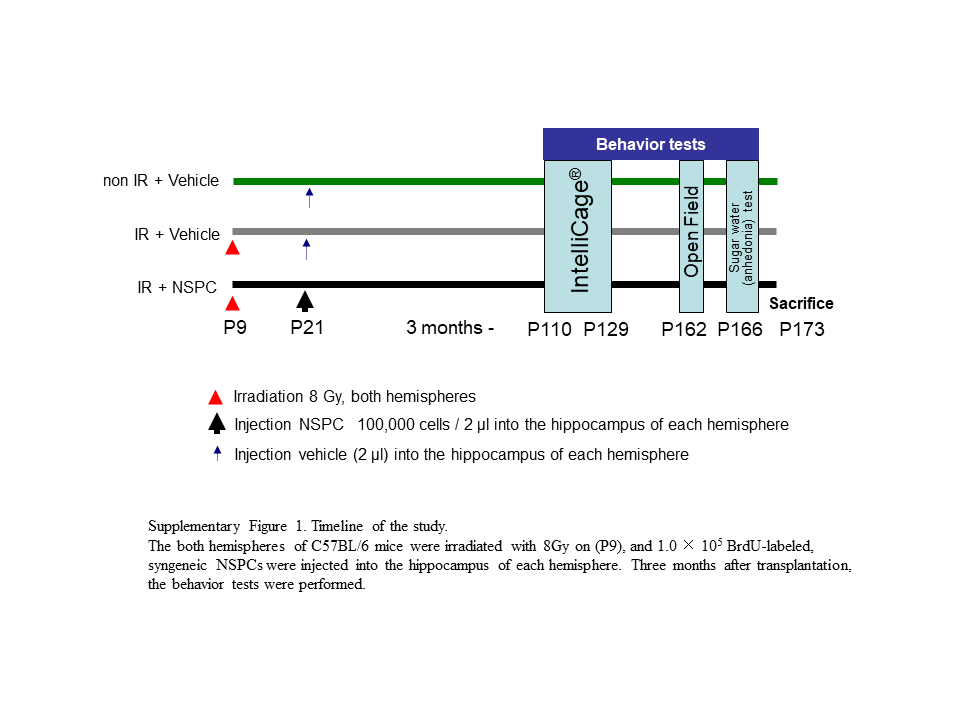

Supplement: Supplementary file 1 [file Image_1.TIF]

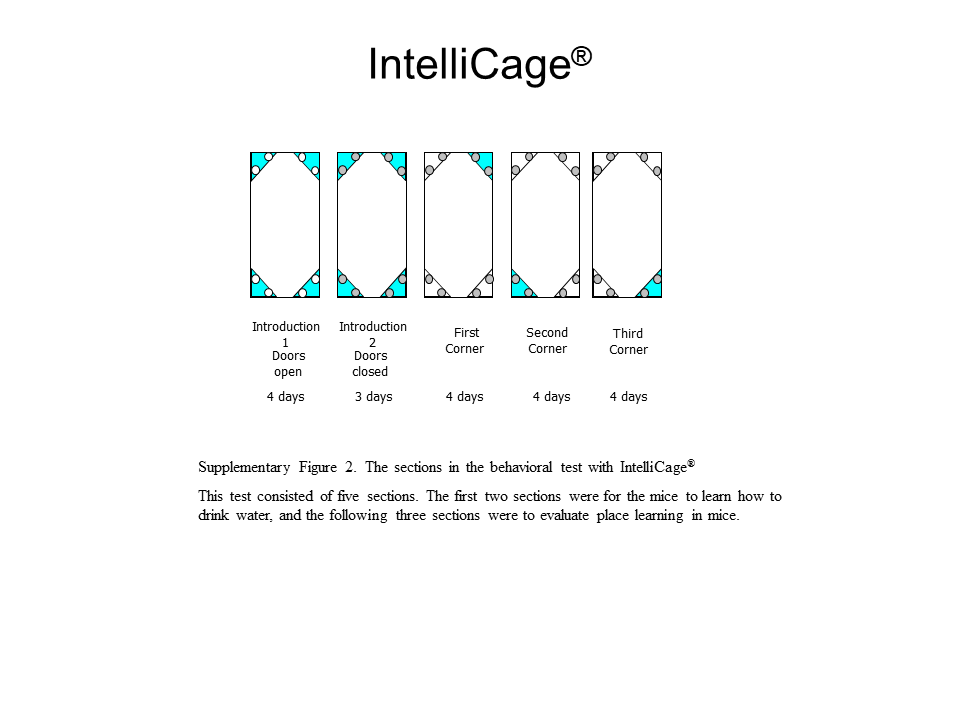

Supplement: Supplementary file 2 [file Image_2.TIF]

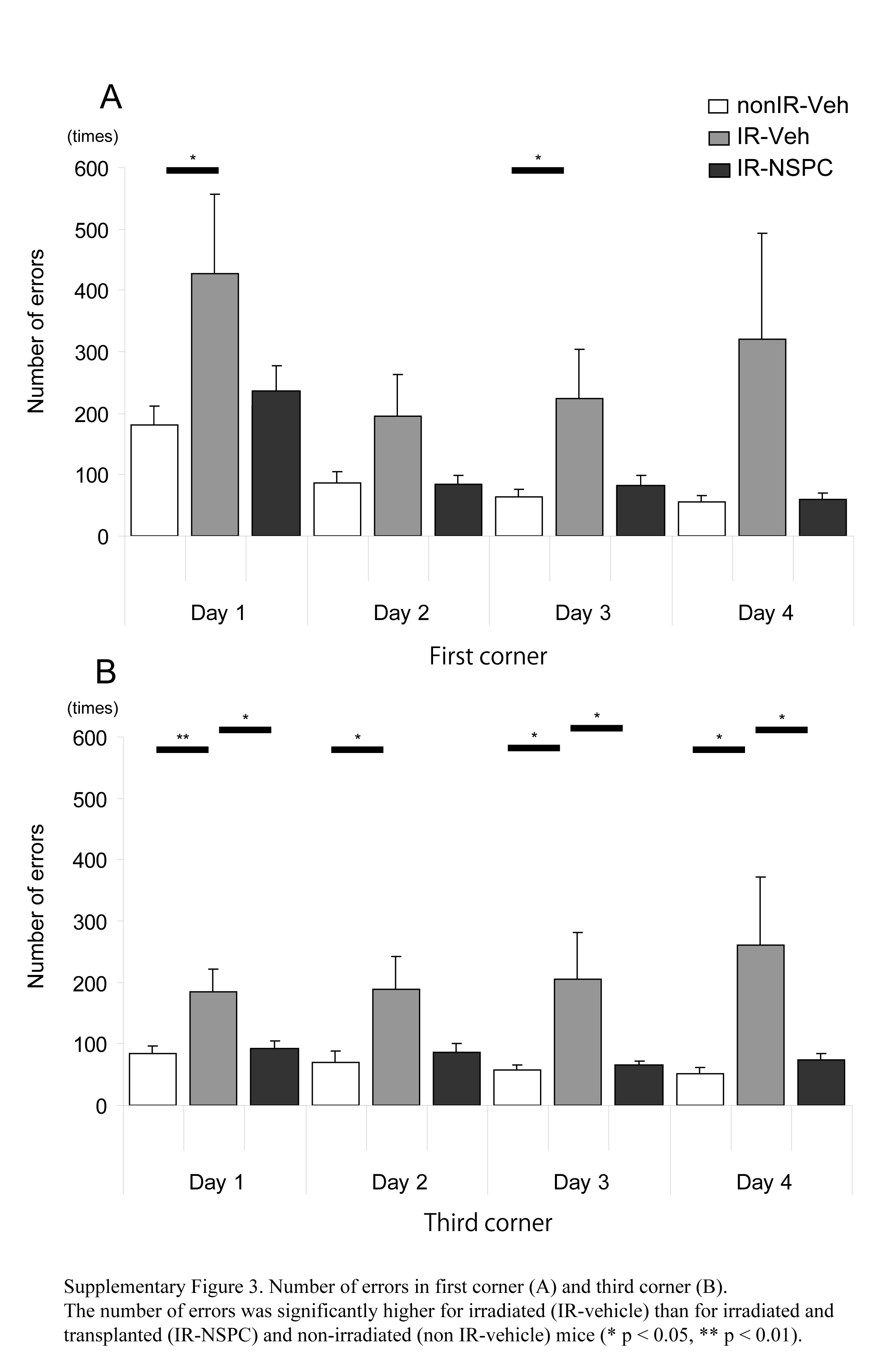

Supplement: Supplementary file 3 [file Image_3.TIF]
